# Characteristics of Stimulant-Induced Psychoses and Schizophrenia Symptoms: A Comparative Analysis Using the PANSS

**DOI:** 10.1192/j.eurpsy.2025.989

**Published:** 2025-08-26

**Authors:** N. Sakels

**Affiliations:** 1 Department of Psychiatry and Narcology, Rīga Stradiņš University, Riga, Latvia

## Abstract

**Introduction:**

The use of stimulants such as amphetamines, cocaine, and methylenedioxymethamphetamine is associated with various mental disorders, including psychoses. These psychotic states can closely resemble the symptoms of schizophrenia, leading to potential misdiagnosis, incorrect treatment, and the assignment of disability status for perceived schizophrenia.

Research indicates that stimulant use can trigger psychoses with characteristics similar to those seen in schizophrenia, including hallucinations, delusions, and disorganized thinking.

**Objectives:**

Investigate the clinical characteristics of stimulant-induced psychoses among patients with stimulant-positive rapid urine test (cocaine, amphetamine, methamphetamine, methylenedioxymethamphetamine, cathinone group) in the Daugavpils Psychoneurological Hospital and the narcology ward of the Daugavpils Regional Hospital.

Assess the clinical presentation of these psychoses in comparison to schizophrenia using the Positive and Negative Syndrome Scale (PANSS), as a validated instrument for evaluating schizophrenia symptoms.

Analyze the data from cases with stimulant-positive rapid urine test and PANSS compare with other research PANSS data in pure schizophrenia psychosis cases.

**Methods:**

1. Data Collection: The study involve participants diagnosed with stimulant-induced psychosis. Data collection will include gathering medical histories with positive stimulant induced psychosis of schizophrenia and stimulant use diagnosis (by ICD-10 F14.5, F15.5, F20+F14/F15) conducting new patient clinical interviews using PANSS, and performing rapid drug urine tests. The Positive and Negative Syndrome Scale will be utilized to quantitatively assess the severity and nature of the participants’ symptoms.

PANSS Scale

Clinically available rapid urine test (MultiDrug Test ect.).

Data Analysis: Clinical data comparisons will be performed using statistical methods.

**Results:**

The comparison of PANSS scores between patients with stimulant-induced psychosis (n=46) and schizophrenia (n=80) is expected to reveal distinct patterns. Patients with stimulant-induced psychosis are likely to exhibit higher scores on the positive symptoms subscale (84%) than in negative (31%), reflecting the more pronounced acute psychotic symptoms triggered by substance use.

In contrast, patients with schizophrenia may exhibit a more balanced profile, with significant impairments in both positive (72%) and negative symptoms (80%).

**Conclusions:**

The clinical presentation of stimulant-induced psychoses can closely resemble that of schizophrenia, leading to diagnostic and therapeutic challenges. This study highlights the differences in PANSS profiles between the two conditions, which can aid clinicians in the differential diagnosis and improve patient outcomes. As well it uncovers the significant impact of rapid urine drug tests in clinical routine.

**Disclosure of Interest:**

None Declared

